# Are T cells the only HIV-1 reservoir?

**DOI:** 10.1186/s12977-016-0323-4

**Published:** 2016-12-20

**Authors:** Abraham Joseph Kandathil, Sho Sugawara, Ashwin Balagopal

**Affiliations:** Department of Medicine, Johns Hopkins University Baltimore, 855 N. Wolfe Street, Rm. 535, Baltimore, MD 21025 USA

**Keywords:** HIV-1, Eradication, Reservoirs, Non-T cells, Challenges

## Abstract

Current antiretroviral therapies have improved the duration and quality of life of people living with HIV-1. However, viral reservoirs impede complete eradication of the virus. Although there are many strategies to eliminate infectious virus, the most actively pursued are latency reversing agents in conjunction with immune modulation. This strategy, known as “shock and kill”, has been tested primarily against the most widely recognized HIV-1 latent reservoir found in resting memory CD4+ T cells. This is in part because of the dearth of conclusive evidence about the existence of non-T cell reservoirs. Studies of non-T cell reservoirs have been difficult to interpret because of technical and biological issues that have hampered a better understanding. This review considers the current knowledge of non-T cell reservoirs, the challenges encountered in a better understanding of these populations, and their implications for HIV-1 cure research.

## Background

In the twenty years since combination antiretroviral therapy (ART) for HIV-1 was first announced, people living with HIV-1 (PLWH) have had marked improvements in mortality and quality of life. However, whereas ART is remarkably effective at preventing new cells from becoming infected, it does not eliminate long-lived cells that are already infected prior to ART initiation. Latent reservoirs have thwarted attempts to eliminate all replication competent forms of the virus from infected individuals [1–6].

There is reason for balanced optimism in the HIV-1 cure field. The ‘Berlin’ and ‘Boston’ patients who underwent bone marrow transplants from donors lacking one or both copies of full-length CCR5, a key HIV-1 entry co-receptor, had prolonged remissions without evidence of HIV-1; in the case of the ‘Berlin’ patient, there is still no evidence of HIV-1 since his transplant [7, 8]. The ‘Mississippi Baby’ and results of the VISCONTI study highlight the possibility of long drug-free remission periods if ART is initiated during primary infection [1, 2, 7, 9–11]. Central to each case of a potential cure or ART-free remission has been a reduction in the size of the HIV-1 reservoir. Therefore, it is critical for cure strategies to target all potential reservoirs.

Many cells are susceptible to HIV-1 in vitro, but not all potential reservoirs have been studied in vivo during ART with the same rigor. Resting memory CD4+ T cells are the most widely recognized and best-described HIV-1 reservoir in research that has been extensively reviewed elsewhere [12, 13]. For cells to constitute an HIV-1 reservoir, they have to harbor replication competent forms of the virus that persist for years despite long-term ART suppression of viremia [14]. Against the standard of the T cell reservoir, in this review we consider evidence suggesting the possible long-term persistence of non-T cell reservoirs in individuals on ART, and the current challenges involved in their identification.

## Usual and unusual suspects

**Fig. 1:**
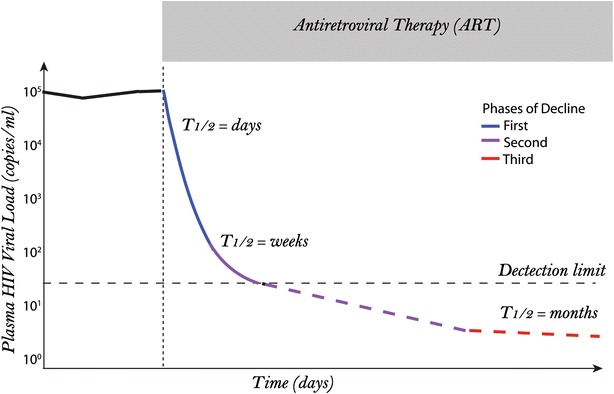
Phasic decline of viremia due to death of HIV-1 infected cells following ART. The multiphasic decay in plasma viremia following initiation of ART has been attributed to the varying half-life of infected cells. Death of productively infected activated CD4+ T cells with a half-life of 1–2 days contributes to the first phase of decline. The slower second phase during which viremia becomes undetectable is contributed to by cells with a half-life in the order of weeks. The cells contributing to the second phase have not been conclusively identified. This is followed by the third phase of decline, characterized by undetectable steady viremia due to infected resting memory CD4+ T cells with a half-life of 44 months

Viral latency is defined as a reversible nonproductive state of infection in individual cells [15]. Reservoirs are cells that harbor replicative forms of HIV-1 following long periods of ART-suppressed viremia [14, 16]. Resting memory CD4+ T cell reservoirs have been estimated to have a half-life of 44 months, meaning that their clearance during ART may take as long as 73 years [13, 17, 18]. Subsequently, distinct populations of CD4+ T cells have also been recognized to contribute to the pool of latently infected cells [19–21], although those are outside the scope of the present review. The half-life of resting memory CD4+ T cell reservoirs corresponds to the long-phase decay of residual plasma viremia in persons taking long-term ART [22]. The phases of plasma HIV-1 RNA decline on ART have been attributed to infection of different cell types that are infected by the virus, and much has been inferred about the identities of those cells without clear evidence (Fig. 1). Here, we enumerate several candidate cell types that could potentially serve as HIV-1 reservoirs (Table 1).

**Table 1:** Summary data on HIV-1 reservoirs and assays in various cell populations

	Memory CD4+ T cells	Myeloid cells		Dendritic cells		FDCs	Epithelial cells
		Monocytes	Macrophages	pDCs	mDCs		
Available VOA?	Yes (gold standard) [23]	Yes [24]	Yes [25]	No	No	Yes [26]	No
Has VOA been applied to PLWH taking long-term ART?	Yes (gold standard) [18]	No	Yes [25]	NA	NA	Yes [26]	No
Has HIV-1 been demonstrated in the indicated cell type in PLWH taking long-term ART?	Yes (gold standard) [18]	No	Yes [25]	NA	NA	Yes [26]	Yes
Is HIV in this reservoir replication competent?	Yes (gold standard) [18]	NA	No	NA	NA	Yes [26]	NA
Available animal models?	Yes [27]	Yes [24]	Yes [24]	Yes [24]	Yes [24]	Yes [27]	No
Have animal models been studied during long-term ART?	Yes [28]	No	No	No	No	No	No
Do animal models with suppressed viremia contain replication competent HIV-1?	Yes [28]	NA	NA	NA	NA	NA	NA
Longevity or T½ of uninfected cells	1–12 months [29, 30]^a^	2–3 days [31]	≥24–36 months [32]^b^	?	?	?	?
Longevity or T½ of reservoir in this cell type	44 months [18]^a^	NA	?	?	?	9 months [33]^c^	?

*?* Not known, *NA* not applicable

^a^There are discrepant data on the longevity of uninfected memory CD4+ T cells and latent HIV-1 reservoirs therein. However, it is difficult to accurately estimate the T_½_ of HIV-1 infected T cells due to possible clonal proliferation: i.e., the listed T_½_ describes the duration of the HIV-1 reservoir itself, but does not directly address the T_½_ of the cell that harbors the reservoir

^b^In the described experiments, donor alveolar macrophages were found 2–3 years after lung transplantation in human subjects: while we assume that these TRM persisted for this duration, it is possible that they underwent proliferation and replacement locally

^c^The indicated longevity is for the infectious virions that were found on FDC dendrites, although it is controversial whether this cell type was actually infected

### Macrophages and myeloid cells

Found primarily in tissues, macrophages are mononuclear leukocytes that are key components of innate immunity. For decades, the origin of tissue resident macrophages (TRM) was explained by the concept of the mononuclear-phagocyte system: monocytes were thought to continually replenish TRM that died in tissues [34, 35]. Consistent with this early concept, the death of HIV-1 infected macrophages was thought to be responsible for the second phase of HIV-1 viral kinetic decline during ART. However, recent findings based on murine models suggest that the principal origin of TRM in steady state is from embryonic haematopoietic precursors, while monocytes only contribute in the setting of inflammation and injury [36]. Similarly, detection of TRM even in individuals with monocytopenia suggests monocyte-independent maintenance, a long half-life of embryonically derived macrophages, or likely a combination of both [37]. Studies in patients who received lung transplantation have also shown long-term persistence of donor alveolar macrophages [32]. In parallel, the rapid second phase decline of HIV-1 was found not to be attributable to macrophages [38]. Taken together, these findings have led to a marked revision in our understanding of the maintenance and longevity of TRM.

It is well established in animal models and in vitro that macrophages can be productively infected by lab strains of HIV-1 [39, 40], although there may be anatomical variation in their susceptibility to HIV-1 infection. For example, there are reports of HIV-1 and SIV in brain macrophages such as microglia [41, 42]. Vaginal macrophages have been shown to support HIV-1 replication better than intestinal macrophages, which may be explained by differential expression of entry co-receptors [43]. Comparative in situ fluorescence also suggests higher HIV-1 susceptibility of rectal macrophages compared to colonic macrophages [44]. Cai et al. have shown that SIV infection of lung macrophages leads to preferential destruction of interstitial macrophages, in comparison to alveolar macrophages that experience minimal cell death and low turnover [45].

Several reports in the pre-ART era demonstrated HIV-1 infection in TRM [46–50]. More recently alveolar macrophages from individuals on ART have been shown to harbor HIV-1 nucleic acids (both proviral DNA and RNA) [51]. Our lab has extended earlier studies of liver macrophages (Kupffer cells), the largest population of TRM in the body, to show that these cells can harbor virus from individuals on ART for as long as 11 years, although their functional significance is still unclear [25]. Other tissue macrophages that have also been implicated as harboring HIV-1 include those in the seminal vesicle, duodenum, urethra, adipose tissue, and liver [25, 46, 52–55].

The study of HIV-1 infection of macrophages is not without controversy. Recent in vivo data from an SIV macaque model has demonstrated the presence of both proviral DNA and T cell receptors (TCR) in myeloid cells: the authors concluded that the presence of viral DNA in macrophages was due to phagocytosis of infected dying cell rather than de novo infection of myeloid cells [56]. However, a subsequent report by Baxter et al. showed that primary monocyte-derived macrophages could selectively capture HIV-1 infected CD4+ T cells, leading to macrophage infection along with efficient HIV-1 cell-to-cell spread [57]. Indeed, others and we have confirmed the exclusion of T cells and TCRs in ex vivo studies of TRM reservoirs [25, 58]. Thus it is important to differentiate between phagocytosis and actual infection of macrophages following detection of nucleic acids in macrophages. In addition, it is clear from in vitro studies that HIV-1 replication dynamics differ in myeloid cells compared to CD4+ T cells: virions can be found dwelling for prolonged periods in long cytoplasmic channels in macrophages and are not immediately released, in contrast to the typical burst that has been described in CD4+ T cells [59].

Monocytes, closely related myeloid cells, were initially reported as being infected in vivo; however, it has now been shown that monocytes are not susceptible to HIV-1, and largely lack proviral HIV-1 DNA in both viremic and ART suppressed individuals [24, 60].

### Dendritic cells

Dendritic cells (DCs) are a heterogeneous group of antigen-presenting cells that play vital roles in orchestrating immune responses [61]. DCs can be broadly divided into those of myeloid or lymphoid origin [62], and further categorized as plasmacytoid (pDCs), myeloid (mDCs), Langerhans cells (found in the epidermis), and interstitial [63].

Although DCs comprise a small proportion of cells in various anatomical sites [64], their role as immunologic sentries makes them among the first cells that encounter invading pathogens like HIV-1. Indeed, analyses of transmitted/founder viruses have shown that they have enhanced binding to mDCs compared to viruses isolated from chronic infection, a feature that may facilitate virus transport across the mucosa [65, 66].

pDCs and mDCs have been noted to have differential susceptibility to HIV-1 infection, although this has largely been ascertained in vitro [67–69]. In vivo, the presence of HIV-1 DNA in DCs has been noted to occur at lower frequency compared to CD4+ T cells [70, 71]. There have been several reports of productive HIV-1 infection of DCs in vitro for as long as 45 days [72–75], but limited data in vivo. Langerhans cells have been considered as a potential reservoir, but largely based on data in the pre-ART era [76, 77].

To fulfill their role as a reservoir, DCs have been posited to transfer infection to T cells, in particular to antigen specific CD4+ T cells, following their encounter with HIV-1, whether or not they themselves are infected [78–80]. This infection in *trans* is mediated by the formation of an infectious/virological synapse [33]. During *trans* infection, compartmentalized HIV-1 has been observed to emerge from DCs and fuse with the T cell membrane [81]. Envelope specific inhibitors maintain their potency against these compartmentalized virions [81]. These are tantalizing hypotheses that have been difficult to find evidence for in vivo.

### Follicular dendritic cells

Follicular dendritic cells (FDCs) that are found in B cell follicles in secondary lymphoid organs are not typical DCs, although they are similarly named: FDCs develop from perivascular precursors of stromal cell origin and are not known to present antigens using MHC-restricted pathways [26, 64].

FDCs can potentially serve as viral reservoirs by maintaining a stable pool of HIV-1 on their surface without being infected [82, 83]. In vitro studies have revealed that HIV-1 virions adhere on the surface of FDCs through interactions with complement receptors mediated via a C3-dependent mechanism [84]. The binding of C3 fragments to the virus allows its adherence to complement receptors CR1 and CR2, present on FDCs [26]. In addition, the presence of non-neutralizing antibodies specific for HIV-1 in patients may enhance binding to FDCs via FcR-mediated binding [26].

HIV-1 has been known to persist on these cells even in the presence of neutralizing antibodies, with reports suggesting that FDCs can restore the infectivity of neutralized viruses [85, 86]. FDCs transfer antigens in the B cell follicles of all secondary lymphoid tissues, and in the process may transfer HIV-1 to T follicular helper cells that are also present in the B cell follicles [21].

In mice, FDCs have been shown to trap HIV-1 following a single exposure, and these virions remained infectious for at least 9 months [85]. A recent study reported visualization of HIV-1 in cycling endosomes in FDCs isolated from individuals on prolonged ART (median = 8 years) [87]. Mathematical models have suggested that FDCs are the major contributor to the low-level viremia detected during the third phase of viral decay, and have been estimated to have a half-life of 39 months [22].

### Epithelial cells

There have been reports suggesting the possible infection and transmission of infection by epithelial cells even though they do not express CD4 and have undetectable or low expression of the co-receptors CCR5and CXCR4 [88, 89]. Renal epithelial cells have been reported to be susceptible to HIV-1 in vitro [90]. Cultures of renal tubule epithelial cells were productively infected by HIV-1 following co-culture with infected T cells [90]. Transmission of infection was observed to occur by formation of virological synapses [91]. HIV-1 mRNA and DNA have also been detected in renal tubular epithelial cells using in situ hybridization done on biopsies obtained from individuals with HIV-1 associated nephropathy [92]. Phylogenetic analyses of sequences obtained from renal epithelial cells were found to cluster together within the radiation of sequences obtained from peripheral blood mononuclear cells [93]. These cells could play a role in persistence of HIV-1 infection in individuals on ART based on indirect evidence [94, 95].

Mammary epithelial cells have been conjectured to harbor a separate compartment of HIV-1: phlyogenetic analyses of HIV-1 DNA from paired breast-milk and peripheral blood samples from HIV-1 infected women have shown the existence of genetically distinct compartments [96, 97]. Studies of breast-milk from HIV-1 infected women on treatment have shown negligible impact of ART on cell-associated or HIV-1 proviral DNA levels, in contrast with a rapid decline in cell-free HIV-1 RNA [98, 99].

Similar to DCs, oral keratinocytes have been shown to support transmission of virus to susceptible cells without supporting replication [100, 101]. However there is no evidence that these cells serve as HIV-1 reservoirs, and there are no published data on the half-life of epithelial cells in vivo in this context.

Kong et al. have reported detection of integrated HIV-1 DNA and release of infectious virus in liver epithelium following in vitro infection of hepatocyte cell lines and primary hepatocytes [102]. In addition, hepatic stellate cells have also been shown to release infectious virus following infection in vitro [103]. However, the translation of this research to studies of in vivo reservoirs has been more challenging, and data are lacking.

### Miscellaneous

There have been isolated reports of other cells that can possibly be infected with HIV-1. Fibrocytes, defined as CD34+CD45+ collagen I+, have recently been reported to have characteristics of cells that can be persistently infected [104]. In vitro, infected fibrocytes resisted HIV-1 induced cell death and stably expressed low levels of HIV-1 mRNA for >60 days. However, there are no data on whether fibrocytes are HIV-1 infected in vivo [104].

Other cell types that could be explored as HIV-1 reservoirs in individuals on ART include astrocytes in the CNS and CD56+/CD3− NK cells [105–107]. Hematopoietic progenitor cells (HPCs) that were initially reported to harbor infectious virus are now not considered to fulfill the criteria to be a reservoir following development of enhanced techniques to purify HSCs from bone marrow [108, 109].

## Challenges in studying non-T cell reservoirs

In ART-suppressed individuals the number of latently infected T cell varies from 1 to 10 infectious units per million (IUPM) [110]. Estimation of these numbers in ART-suppressed individuals requires isolation of millions of cells from large volume blood draws [111]. Similar studies on cells from HIV-1 infected people that have low or absent numbers in circulation, or that are principally found in tissues, have been technically challenging or unethical [25, 51].

## Technical challenges

The gold standard for quantifying the amount of replication competent HIV-1 in a purified population of cells during ART has been the quantitative viral outgrowth assay (QVOA), which was initially developed to measure the amount of latent HIV-1 infection in resting memory CD4+ T cells [23, 112]. The potency of the QVOA is that it hinges on the recovery of infectious, replication competent HIV-1 that propagates exponentially, plausibly explaining the virological rebound seen in patients who discontinue ART. The QVOA is a highly consistent assay, but nonetheless poses a number of technical challenges, including that it is expensive, time-consuming, requires large amounts of starting materials, has a limited dynamic range, and underestimates the size of the latent reservoir [111–113]. Several groups have employed PCR-based approaches as alternative tools [23]. PCR-based assays sensitively detect viral nucleic acid over a large dynamic range, and can differentiate between total, integrated, and LTR HIV-1 DNA [114, 115]. Although easier, PCR-based approaches do not differentiate between replication competent and defective viruses, of which the latter constitute the majority of viral forms, and do not correlate well with the number of cells with replication competent virus [13]. PCR-based approaches typically yield infected cell frequencies that are 100–1000 times higher than what is resulted from the QVOA [23]. More recently, an approach called the TILDA (*Tat*/*rev* Induced Limiting Dilution Assay) that measures multiply spliced HIV-1 RNA was developed as an alternative [116]. This assay has a quick turnaround time and requires fewer than a million cells of starting material. However, the TILDA does not measure virus production and does not address whether measured RNAs derive from replication competent viruses [116, 117]. Moreover, the TILDA correlates poorly with the QVOA when performed on the same samples [116].

Therefore, as of now the most accurate measurement of the replication competent viral reservoir requires the QVOA, limiting the quantification of HIV-1 reservoirs in tissues that are poorly accessible. However, an overlooked challenge of using the QVOA is that it has been specifically “tuned” to CD4+ T cells, and may not be sensitive for detecting infection in cells that bear different HIV-1 replication dynamics than CD4+ T cells. Recent advances in adapting the QVOA to macrophages are steps in the right direction for quantifying these HIV-1 reservoirs [58].

## Biologic solutions

To address the challenges posed in isolating a large number of these cells to study latency, the field has resorted to the use of alternate models that complement each other—in vitro, animal, and mathematical models [22, 58, 118, 119]. Although more feasible, these approaches have their drawbacks. In vitro models are used frequently because of their convenience, but do not fully mimic in vivo infections. [64, 120]. Similarly, heterogeneous cell phenotypes can be observed in in vitro models, such as in monocyte-derived macrophages (MDMs) subpopulations [121–123]. Fundamentally, HIV-1 susceptibility and longevity in vitro may be quite different than in the immunological context of natural infection. Hence, in vitro modeling can only be used to complement findings in vivo.

Non-human primates (NHP) and humanized mice models have been invaluable for understanding HIV-1 pathogenesis [24, 27, 58]. NHP are typically infected either with simian immunodeficiency virus (SIV) or SIV/HIV-1 chimeric viruses (SHIV) [27, 124]. However, SIV and HIV-1 have notable distinctions, sharing only approximately 53% sequence homology and differing in the organization of their overlapping ORFs [124]. For instance, sooty mangabey SIV (SIVsmm) and macaque SIV (SIVmac) lack the HIV-1 accessory gene *vpu*. Instead, they encode for *vpx*, which may be a critical difference: *vpx* degrades SAM and HD domain containing deoxynucleoside triphosphate triphosphohydrolase 1 (SAMHD1), a key retroviral restriction factor in macrophages and DCs [125, 126]. Nevertheless, SIV infection of NHP remains a key experimental tool, especially for in vivo and ex vivo studies of tissues that are inaccessible in humans, such as the brain.

Recent advances in humanized mouse technology have facilitated their infection with HIV-1 [127–129]. A recent humanized model referred to as myeloid-only-mice (MoM), developed from NOD/SCID mice, has been very useful to study infection and persistence in non-T cells [24, 130]. These mice lack T cells, and are developed by adoptive transfer of human CD34+ stem cells, enabling reconstitution of the mouse with human monocytes, macrophages, B cells, and dendritic cells [24, 130]. However, a major hurdle impeding more widespread use of humanized mice is that each experiment requires the surgical engraftment of human tissue, since this aspect cannot be bred [124]. A promising and creative use of humanized mice is in the development of a murine viral outgrowth assay where HIV-1 latency is estimated by adoptive transfer of human cells into humanized mice [131].

## Conclusion

Whereas promising improvements to antiretroviral therapy have improved the quality of life of PLWH, they have not bridged the gap toward an HIV-1 cure [132]. Although it has been debated whether resources for HIV-1 research should be focused on a cure when there are other challenges facing PLWH, we argue that latent reservoirs harbor the potential for high-level virologic rebound in each of the 37 million HIV-1 infected people worldwide, which bears both individual and public harm. Indeed, we further argue that without exploring the true extent of HIV-1 reservoirs with the same rigor as has been used to study peripheral resting memory CD4+ T cells, we risk developing incomplete cure strategies [18, 110]. The current “shock and kill” strategy hinges on the drugs known as latency reversing agents (LRAs) that induce viral production in latently-infected cells [13, 133–135]. Presently, however, latency reversal has been developed to be specific for CD4+ T cell biology, and does not account for the possibility of persistent reservoirs in cells other than T cells [136, 137], reflecting lacunae in our understanding of non-T cell reservoirs [28]. Therefore, a dedicated strategy to eliminate HIV-1 reservoirs requires a better understanding of the role of non-T cell reservoirs using in vivo and ex vivo experimentation.

## Abbreviations

ART: antiretroviral therapy
PLWH: people living with HIV-1
TRM: tissue resident macrophages
TCR: T cell receptors
DCs: dendritic cells
pDCs: plasmacytoid dendritic cells
mDCs: myeloid dendritic cells
FDCs: follicular dendritic cells
HPCs: hematopoietic progenitor cells
IUPM: infectious units per million
QVOA: quantitative viral outgrowth assay
TILDA: *Tat*/*rev* Induced Limiting Dilution Assay
MDMs: monocyte-derived macrophages
NHP: non-human primates
SIV: simian immunodeficiency virus
SHIV: SIV/HIV-1 chimeric viruses
SIVsmm: sooty mangabey SIV
SIVmac: macaque SIV
SAMHD1: SAM and HD domain containing deoxynucleoside triphosphate triphosphohydrolase 1
MoM: myeloid-only-mice
LRAs: latency reversing agents
