# Erratum to: Are T cells the only HIV-1 reservoir?

**DOI:** 10.1186/s12977-017-0333-x

**Published:** 2017-02-08

**Authors:** Abraham Joseph Kandathil, Sho Sugawara, Ashwin Balagopal

**Affiliations:** Department of Medicine, Johns Hopkins University Baltimore, 855 N. Wolfe Street, Rm. 535, Baltimore, MD 21025 USA

## Erratum to: Retrovirology (2016) 13:86 DOI 10.1186/s12977-016-0323-4

Unfortunately, the original version of this article [1] contained an error. Table 1 has errors with the references. The correct Table 1 is found below.

**Table 1:** Summary data on HIV-1 reservoirs and assays in various cell populations

	Memory CD4+T cells	Myeloid cells		Dendritic cells		FDCs	Epithelial cells
		Monocytes	Macrophages	pDCs	mDCs		
Available VOA?	Yes (gold standard) [112]	Yes [24]	Yes [25]	No	No	Yes [87]	No
Has VOA been applied to PLWH taking long-term ART?	Yes (gold standard) [18]	No [24]	Yes [25]	NA	NA	Yes [87]	No
Has HIV-1 been demonstrated in the indicated cell type in PLWH taking long-term ART?	Yes (gold standard) [18]	No [24]	Yes [25]	NA	NA	Yes [87]	Yes [98, 99]
Is HIV in this reservoir replication competent?	Yes (gold standard) [18]	NA	No	NA	NA	Yes [87]	NA
Available animal models?	Yes [124]	Yes [24, 58]	Yes [24, 58]	Yes [130]	Yes [130]	Yes [85]	No
Have animal models been studied during long-term ART?	Yes [138]	No	No	No	No	No	No
Do animal models with suppressed viremia contain replication competent HIV-1?	Yes [138]	NA	NA	NA	NA	NA	NA
Longevity or T½ of uninfected cells	1–12 months [29, 30]^a^	2–3 days [31]	≥24–36 months [32]^b^	?	?	?	?
Longevity or T½ of reservoir in this cell type	44 months [18]^a^	NA	?	?	?	9 months [85]^c^	?

? Not known, *NA* not applicable

^a^There are discrepant data on the longevity of uninfected memory CD4+T cells and latent HIV-1 reservoirs therein. However, it is difficult to accurately estimate the T_½_ of HIV-1 infected T cells due to possible clonal proliferation: i.e., the listed T_½_ describes the duration of the HIV-1 reservoir itself, but does not directly address the T_½_ of the cell that harbors the reservoir

^b^In the described experiments, donor alveolar macrophages were found 2–3 years after lung transplantation in human subjects: while we assume that these TRM persisted for this duration, it is possible that they underwent proliferation and replacement locally

^c^The indicated longevity is for the infectious virions that were found on FDC dendrites, although it is controversial whether this cell type was actually infected

^138^ Dinoso JB, Rabi SA, Blankson JN, Gama L, Mankowski JL, Siliciano RF, Zink MC, Clements JE. A simian immunodeficiency virus-infected macaque model to study viral reservoirs that persist during highly active antiretroviral therapy. J Virol. 2009;83(18):9247–57
